# Assessing Impacts of Transgenic Plants on Soil Using Functional Indicators: Twenty Years of Research and Perspectives

**DOI:** 10.3390/plants11182439

**Published:** 2022-09-19

**Authors:** Vadim Lebedev, Tatyana Lebedeva, Elena Tikhonova, Konstantin Shestibratov

**Affiliations:** 1 Branch of the Shemyakin-Ovchinnikov Institute of Bioorganic Chemistry of the Russian Academy of Sciences, Prospekt Nauki 6, 142290 Pushchino, Russia; 2 Institute of Physicochemical and Biological Problems in Soil Science of the Russian Academy of Sciences, Instituskaya Str. 2, 142290 Pushchino, Russia; 3 Department of Landscape Architecture and Soil Science, Voronezh State University of Forestry and Technologies Named after G.F. Morozov, 8 Timiryazeva Str., 394087 Voronezh, Russia; 4 Pushchino State Institute of Natural Sciences, Prospekt Nauki 3, Pushchino, 142290 Moscow, Russia

**Keywords:** biosafety, transgenic plants, risk assessment, soil enzyme activity, microbial biomass, soil fertility

## Abstract

Assessment of the effects of transgenic plants on microbiota and soil fertility is an important part of the overall assessment of their biosafety. However, the environmental risk assessment of genetically modified plants has long been focused on the aboveground effects. In this review, we discuss the results of two decades of research on the impact of transgenic plants on the physicochemical properties of soil, its enzyme activities and microbial biomass. These indicators allow us to assess both the short-term effects and long-term effects of cultivating transgenic plants. Most studies have shown that the effect of transgenic plants on the soil is temporary and inconsistent. Moreover, many other factors, such as the site location, weather conditions, varietal differences and management system, have a greater impact on soil quality than the transgenic status of the plants. In addition to the effects of transgenic crop cultivation, the review also considers the effects of transgenic plant residues on soil processes, and discusses the future prospects for studying the impact of genetically modified plants on soil ecosystems.

## 1. Introduction

Socioeconomic benefits of genetically modified plants (GMP) have favored their wide cultivation in the world so that most cotton (79%) and soybean (74%), as well as a significant part of maize (31%) and canola (27%), are biotech crops [1]. The emergence of transgenic plants has raised a debate about their biosafety, both for human health and the environment. Soon after the commercial release of the first genetically altered crop in 1996, Snow and Moran-Palma [2] divided environmental risks into four groups: (1) transgene flow to wild relatives, (2) the evolution of resistant pests, (3) the effects on non-target organisms and (4) the effects on soil biota and fertility. Therefore, assessment of the risks to soil due to the cultivation of GMPs should be an important part of an overall safety assessment of transgenic plants. However, the environmental risk assessment of transgenic plants has long been focused mainly on the aboveground effects. In the early 2000s, several reviews summarized the research on transgene flow to wild and/or weedy relatives [3], the evolution of resistant pests [4] or the effects on non-target organisms [5]. At the same time, studies on the effects of transgenic plants on soil microorganisms were just beginning. Currently, this aspect is very important because microorganisms are the dominant underground soil organisms: they account for more than 80% of the total biomass (without roots) and largely determine the functioning of terrestrial ecosystems [6]. The disregard for the effects of transgenic plants on the underground components is mainly explained by the difficulties inherent in the study of soil microbiota [7]. The abundance of various biotic and abiotic factors has so far prevented researchers from reaching a consensus on whether GMPs can affect soil microorganisms [8]. The available studies provide contradictory results. Most studies have assessed the effects as insignificant, although some studies have noted significant effects, although these are transitory. On the other hand, there are known publications on the negative effects of transgenic plants on the physical and chemical properties of soil, its microbial biomass, enzyme activities and microbial biodiversity [9,10,11]. Our review summarizes two decades of research on the effects of GMPs on the physicochemical properties of soil, its enzyme activities and microbial biomass. We also note the involvement of external factors in these interactions and outline the prospects for further research on these issues.

## 2. Potential Risks of Transgenic Plants for Soil Ecosystems

**Figure 1:**
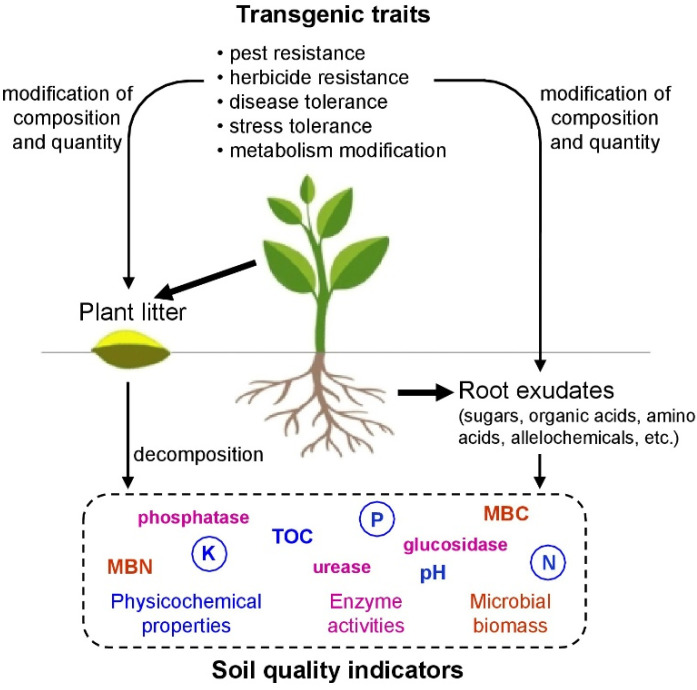
Potential impact of transgenic plants on soil quality indicators.

Soil microorganisms are essential components of soil biological activity and are involved in important biochemical processes such as the decomposition of organic matter, humus formation and the transformation and cycling of nutrients [12]. Rhizosphere, the root–soil interface, is the key site where microorganisms integrate soil and plants [8]. Plants release up to 20% of the photosynthetically fixed carbon into soil with root exudates, which are the main source of C for microorganisms [13]. These exudates contain primary and secondary metabolites and, via solubilization and mineral desorption, provide microorganisms with nutrients, thus playing a key role in establishing plant–microorganism interactions [14]. Therefore, any intended or unintended alteration in the composition or quantity of root exudates can affect the soil microbiota (Figure 1). In addition to the well-known components (sugars, organic acids, amino acids, etc.), the root exudates of transgenic plants can also contain new substances, including toxins [15]. Microorganisms also use plant residues—shoots and roots—as a source of C and nutrients. There is no clear understanding of what is the cause of changes in nutrient cycling in soil under GM crops— differences in the composition and quantity of root exudates or plant residues (proteins, carbohydrates, lignins, etc.) [16].

Different authors differently define the potential effects of GMPs on soil [15,16], but generally these effects can be divided into three groups: (1) direct effects via new GMP-produced substances, e.g., Bacillus thuringiensis (Bt) toxins; (2) indirect effects due to intended or unintended changes in plant metabolism that alter the quality and quantity of root exudates or the composition and quantity of biomass, mostly underground; (3) changes in management systems associated with the introduction of GM crops, e.g., the use of other herbicides. Any of them can boost or inhibit the growth of certain groups of microorganisms and thus may ultimately affect the processes of carbon and nutrient cycling on the ecosystem level [17]. At the very beginning of research in this area, however, the question was asked as to whether a transgenic plant with as little as one or two genes that made it differ from the original plant could have significant effects on soil microorganisms [18]. It looks more likely that fluctuations in temperature or precipitation, or changes in plant management would have a much greater impact on such a heterogeneous system as soil than an altered genetic trait could [16]. An accurate assessment of changes in soil ecosystem requires selecting the proper indicators.

## 3. Indicators of Soil Quality and Fertility

In the extremely complex “plant–soil–microbes” system, each component should be evaluated with its specific dedicated methods. Most often, a transgenic plant is already well characterized by the beginning of experiments, and the only task is to ascertain some parameters under specific growing conditions. Soil quality research traditionally focused on chemical and physical properties [19]. The number of such parameters is quite limited (pH, organic matter, macro- and some micronutrients) and they can be determined by simple analytical methods. A wide variety of methods assess soil microorganisms, their diversity, abundance and function. Microbial diversity is studied using molecular tools, particularly amplicon sequencing, terminal restriction fragment length polymorphism (T-RFLP) and denaturing gradient gel electrophoresis (DGGE), while the phospholipid fatty acids (PLFA) analysis provides information about the overall structure of a microbial community [20]. The total microbial biomass is assessed by quantifying the biophilic elements in microbial cells, most often C (microbial biomass C, MBC), less often N (microbial biomass N, MBN) and even less often P (microbial biomass P, MBP). The functionality is evaluated by measuring the activities of soil enzymes, which are very diverse.

Great attention is given to the effects of GMPs on biodiversity. The review by Guan et al. [8] provides a detailed discussion of the effects of transgenic plants on the soil microbial diversity as assessed using PLFA, DGGE, T-RFLP and other methods. Understanding the relationship between microbial composition and functionality is necessary to predict changes in ecosystem functioning in response to various environmental disturbances [21], including the impact of transgenic plants. However, relationships between microbial diversity and soil functions are still debated [22]. The concept that biodiversity promotes the functioning of ecosystems has long been adopted for animals and plants. If directly extended to microorganisms, however, the concept faces a number of serious issues [23]. These issues originate from fundamental differences between macro- and microorganisms: the latter are characterized by small sizes but an immense richness of microbes, faster metabolism and physiological versatility, and rapid and colonial growth [24,25]. There is no direct evidence that the microbial diversity of soil is related to soil ecosystem functioning. The existing methods and techniques are either not effective enough to obtain relevant evidence, or the available data may be insufficient for valid conclusions [8]. A recent study was the first to compare five levels of soil microbial diversities of taxonomy and function responding to biodiversity loss based on global soil metagenomes across diverse biomes [26]. It showed that the relative abundance of microbial functioning can remain stable despite a sharp reduction in taxonomic species that leads to biotic homogenization but functional stability. This stability suggests a decoupling of taxonomy and function. The cause of this stability is that microbial communities have high taxonomic variability but a stable functional structure [27]. Assuming that changes in functionality are more likely to be the consequence of diversity disturbance than vice versa, we focused on the analysis of GMP effects on the physicochemical properties of soil, its enzyme activities and microbial biomass.

### 3.1. Soil Physicochemical Properties

The composition of a soil microbial community is the result of the soil’s physical and chemical properties, which develop at different time scales, over a long period of soil formation, as well as more recent, in response to local weather conditions and management systems [28]. For instance, pH is one of the most powerful factors that affects the composition of a soil microbial community [29]. Another example is the relationship between the electrical conductivity (EC) of soil and its microbial biomass [30]. Thus, changes in soil’s physicochemical properties can directly or indirectly affect the activities of soil enzymes and its microbial biomass [31]. Plant roots and soil microbes can, in turn, alter the physical and chemical properties of soil in the rhizosphere [32]. For example, organic acids from root exudates not only alter pH but also play an important part in the availability of phosphorus. Changes in the composition and quantity of root exudates in plants with new genetic traits may have a direct effect on transformations of soil P and/or an indirect effect on the availability of P via shifts in the microbial community and the activities of microorganisms inhabiting the rhizosphere [16].

### 3.2. Soil Enzyme Activity

The enzymatic activity of soil plays a crucial role in the formation and decomposition of soil organic matter, as well as in nutrient cycling [33]. In many studies, soil enzymes are used as indicators of microbial activity and soil fertility. Their activity is considered an early and sensitive indicator of natural or anthropogenic disturbances [34]. The list of microbial genotypic function traits important to biogeochemistry, ecology and environmental sciences includes enzymes such as chitinase xylanase (carbon degradation in carbon cycling), urease (N mineralization in N cycling), etc. [23].

The most important enzymes associated with changes in soil quality are hydrolases and oxidoreductases. The best studied ones include the intracellular enzyme dehydrogenase (DHA), an oxidoreductase, and a number of extracellular hydrolases (β-glucosidase (BGL), phosphatase (PHO), urease, arylsulfatase (ARS) and others), which are directly involved in transformations of organic compounds and the release of C and nutrients, such as N, P and S [35]. The hydrolytic degradation of complex soil components is an important step in several biogeochemical cycles [36]. The thus obtained carbon and nutrients are then assimilated by microbial cells and used in several metabolic pathways controlled by intracellular enzymes, including DHA. Even before studies with transgenic plants, soil quality was often assessed based on enzyme activities, such as DHA (general biochemical parameter), PHO, BGL and urease (P, C and N cycles) [37]. The same enzymes, as well as protease and arylsulfatase, are most often used to assess the effects of transgenic plants on soil microorganisms. The DHA activity is an important indicator of oxidative metabolism in soils and a sensitive marker of soil microbial activity since this intracellular enzyme is associated with viable cells [38]. β-glucosidase catalyzes cellobiose hydrolysis to glucose and dominates among other enzymes involved in the degradation of carbohydrates in soils [39]. Urease plays an essential role in the effective use of urea in soil by hydrolyzing it to NH_3_ and CO_2_; changes in urease activity are an indirect indicator of changes in the pool of potentially available N in soil [40]. Protease is another essential enzyme in the N cycle in soil; it breaks proteins down to amino acids and is often considered to be the rate-limiting step of N mineralization [41]. Phosphatases catalyze the cycling and transformation of P in soil ecosystems, and they are a good indicator of organic P mineralization and soil activity [42]. Most often, studies assess the activities of acid PHO, which is mainly produced by plant roots, and alkaline PHO, which originates from microorganisms and fauna [43]. Arylsulfatase is an important soil enzyme catalyzing the hydrolysis of sulfate esters [44].

Less often assessed are hydrolases, such as cellulase and invertase, which hydrolyze cellulose and sucrose to monosaccharides, respectively [45,46], and oxidoreductases, such as polyphenol oxidase and catalase, which degrade recalcitrant aromatic compounds and hydrogen peroxide, respectively [47,48].

### 3.3. Microbial Biomass

Soil microbial biomass is a critical component of most terrestrial ecosystems because it regulates nutrient cycling and acts as a highly labile source of nutrients available to plants [49]. For instance, MBC is more sensitive to changes in the status of organic matter than the total organic C is [50]. MBC, MBN and MBP are the active components of C, N and P in soil, respectively, and, as such, participate in the cycling of these elements in the ecosystems [51]. MBC is the driving force of the decomposition of soil organic matter, while MBN is critical to regulating the N flow into soil. MBP governs the mineralization and fixation of soil P, reflects the capacity and intensity of its cycling and is an important source of available soil P [11].

The microbiological parameters of soil (enzyme activities and biomass) are considered more sensitive to changes in management and environmental conditions than chemical or physical properties are [7,52]. For example, TOC is relatively insensitive to environmental changes and reflects the cumulative result of changes in affecting factors over a relatively long period [53]. Thus, biochemical indicators show the early response of soil to exposure, while physicochemical indicators reflect longer-term trends. Together, they complement each other and provide information about the intensity and direction of changes in a soil ecosystem.

## 4. Effects of Transgenic Plants’ Cultivation

According to recent data, the most cultivated transgenic plants in the world in 2019 were those with stacked traits with insect resistance and herbicide tolerance, and herbicide-tolerant and insect-resistant crops, which occupied 45%, 43% and 12% of the global biotech crop area, respectively [1]. All other GM crops—virus-resistant or salt-tolerant plants, canola with modified oils, low gossypol cotton, etc.—accounted for less than 0.5% of the global biotech crop area. Despite the dominance of herbicide-resistant crops, most studies on the GMP effects on soil were conducted with insect-resistant plants because they produce toxic Bt proteins (Table 1). Plants with other traits are studied less frequently, although theoretically they may also cause unintended changes unrelated to the new gene product but able to affect soil processes.

### 4.1. Insect-Resistant Transgenic Plants

Pot studies of Bt cotton (*Cry1Ac*) in India found no differences in NH4 [58] and TOC [59] in soil. However, Bt cotton showed significant variations in available P, with the availability levels both lower (in mid-vegetation) and higher (at the end of vegetation) versus a control [58]. In another pot experiment conducted in China, Bt cotton with the same gene (*Cry1Ac*) had no effect on the content of organic matter, total N, available N or K throughout the growing period [63]. The study also noted a significant decrease in available P in Bt cotton, although only during a flowering period.

According to a later short-term field study, Bt cotton did not show any significant adverse effects on the physicochemical properties of soil as compared with non-transgenic plants. Indian studies did not reveal any significant effect of Bt cotton on the content of available N, P and K [71]. Studies in Pakistan showed that Bt cotton had no effect on pH and EC, while its effects on other parameters depended on NPK fertilizers [32]. TOC did not differ among treatments without fertilizers, but was significantly higher in two clones in treatments with fertilizers. Phosphorus, conversely, did not differ among treatments with fertilizers, but was significantly higher in all four clones in the presence of fertilizers. The content of available K in various clones, regardless of fertilizers, could be either higher or lower than in the control [32].

The long-term field tests of Bt plants had ambiguous results. Field cultivation of Bt maize for 7 years did not significantly alter the total C or total N, or the soil texture [60]. An 8-year cultivation of Bt rice did not bring any consistent changes in soil properties: the control samples contained significantly less TOC and TN mid-season and significantly less available P at the end of the season [53]. Long-term field tests of Bt poplar showed no effect on the content of N [11,70]. The effect on P, however, differed: while the Bt poplar showed no effect in one study [70], all five Bt poplar lines significantly reduced the content of available P in another [11]. The authors suggest that transgenic trees had a negative impact on the activity of phosphate solubilizing microorganisms and thus affected the transformation of soil P. On the whole, cultivation of Bt plants did not change the physical and chemical properties of soil; however, many researchers noted their influence on the content of available P.

One- to two-year field tests revealed no effect on soil enzyme activities in crops such as maize [57], sugarcane [68] or cotton [12]. In general, after a 4-year cultivation in the field, there were no consistent significant differences in the activities of N-, P- and S-cycle enzymes and DHA in maize with *Cry1Ab* or *Cry3Bb1* genes and a non-transgenic control [55]. Random significant differences were not stable and did not persist. A number of studies reported the effect of Bt plants on individual enzymes. For example, acid and alkaline PHOs were significantly increased in Bt cotton pots by the end of cultivation [59]. A 3-year cultivation of Bt cotton in the field had no effect on the activities of extracellular enzymes, whereas the DHA activity increased significantly [61]. According to another report, three field-grown Bt cotton clones significantly increased the urease and DHA activities [40]. The authors believe that the increase in the DHA activity could be due to a higher microbial activity stimulated by the increased root density in Bt cotton compared with the control. On the other hand, field-grown Bt maize had no effect on urease but significantly reduced the activities of BGL and acidic PHOs [69]. This indicates that some bacterial species could have been inhibited and did not participate in the metabolic activity of soil.

Unlike many studies, Chen et al. [9] showed an inhibitory effect of cotton with pest resistance genes, when cultivated in pots in the greenhouse, on enzymes of the N, P and S cycles, as well as DHA and catalases, with the BGL activity being the only one unchanged. The authors explain the discrepancy in findings by the absence or too low levels of Cry proteins in the soils of previous researchers. They attribute the reduction in enzyme activities in the soil of transgenic cotton to decreased enzyme synthesis by microorganisms or to a competition between the Cry1Ac proteins and the CpTI enzymes for adsorption sites in soil. A subsequent field assessment showed a significant increase in the DHA activity in soil growing Bt cotton and its significant inhibition after residue incorporation in soil [10]. Thus, similarly to its effects in the greenhouse, Bt cotton inhibited the growth and activities of soil microorganisms. The observed stimulation of extracellular enzymes could have been associated with the adsorption of Cry1Ac proteins on soil particles, the release of a certain amount of enzymes and the increase in their activities. The increased enzyme activities can accelerate the C, N, P and S cycles in soil and should therefore be considered as a potential unintended risk of transgenic Bt cotton associated with adding its residues into soil [10].

Bt plants had ambiguous effects on soil microbial biomass. Devare et al. [17,54] reported the absence of any significant effect of Bt maize with the *Cry3Bb* gene on MBC after two or three years of field tests in the USA. Five-year tests of Bt maize with the *Cry1Ab* gene also showed no effect on MBC [66], the same as 2-year tests of Bt cotton [12]. Nor were there any differences in MBC and MBN after 8 years of growing Bt rice in the field [53]. On the other hand, there were reports of microbial biomass stimulation in soil from Bt cotton. Significantly higher values of MBC, MBN and MBP were found in soil from Bt cotton grown in pots under net house conditions [59]. Field tests also confirmed the stimulating effect of three Bt cotton lines on MBC in a layer of 0 to 15 cm, but only for one line in a layer of 15 to 30 cm [40]. A significant increase in MBC in the field in one of four Bt cotton lines, both with and without fertilizer, suggests that it was peculiar to this specific transgenic genotype [32].

In contrast to those results, three and four years of greenhouse pot cultivation of Bt and Bt+CpTI cotton resulted in a significant reduction in MBC, which indicates the inhibition of microbial activity in the soil of transgenic plants [9]. Further field studies confirmed the significant inhibition of MBC by Bt cotton plants [10]. Four-year field tests of Bt poplar in China showed a significant effect on soil microbial biomass: in soil samples from all five clones, MBC was significantly higher, while MBN and MBP were significantly lower, compared with the control [11]. These changes modify the ability of soil microorganisms to metabolize C, N and P and thus can ultimately affect the plant growth. The lower MBN and MBP in the soil from Bt poplars indicates that soil microorganisms are stressed by nutrient deficiencies. Moreover, there were also changes in the structure of the soil microbial community. In a control, the MBC/MBN ratio was about 4.6, which indicates the dominant role of bacteria; in Bt poplars, it was about 9.2, showing the predominance of fungi [11].

### 4.2. Herbicide-Resistant Transgenic Plants

The cultivation of herbicide-resistant plants (Table 2) does not imply an a priori effect on soil microflora, and a greenhouse-grown oilseed rape with the *pat* gene did not affect ARS or MBN, although there were significant changes in the activities of invertase, phosphatase and urease [72]. The latter were probably caused by changes in the composition and/or concentration of root exudate due to unintended alterations in the transformation process. The change in exudation is also evidenced by a high invertase activity in the rhizospheres of senescent transgenic plants, which is indicative of increased sucrose concentrations in the root zone [72].

Although herbicide-resistant plants occupy the largest part of GM crop areas, there have not been many studies with them. This fact, however, is offset by the large scale of those studies. Field tests in Canada and Brazil lasted for 3 to 9 years, were carried out on several sites with different soil and climatic conditions, and included various management systems and crop rotations (Table 2). In those studies, GMPs resistant to glyphosate or imidazolinone showed no significant effect on MBC, MBN [73,74,75,76] or soil physicochemical properties (Ca, Mg, K, organic matter, N, P, cation exchange capacity, Mn, Fe, Cu, Zn, soil density and granulometry), except for pH [76] or enzymatic activity [73,74,77].

**Table 2:** Risk assessment of herbicide-resistant transgenic plants on soil quality.

Species	Gene	Growth Conditions	Indicators	Additional Factors	References
oilseed rape	*pat*	greenhouse	MBN	growth stage	[72]
			C, N, P, S		
wheat	*epsps*	field (4 years)	MBC	location	[73]
canola			DHA	crop rotation	
maize	*epsps*	field (5 years)	MBC	herbicide	[74]
			C	crop rotation	
soybean	*ahas*	field (3 years)	MBC, MBN	location	[75]
soybean	*epsps*	field (3 years)	MBC, MBN	location	[77]
			C, P	herbicide	
soybean	*epsps*	field (8–9 years)	pH, org. matter, N, P, microelem., texture	location	[76]
			MBC, MBN		

### 4.3. Disease-Tolerant Transgenic Plants

One of the first disease-tolerant transgenic plants authorized for commercial use back in 1996 was virus-tolerant papaya expressing the coat protein gene of the Papaya ringspot virus (PRSV) (Table 3). Studies on pot-grown papaya did not reveal any effect on pH, organic matter, P, K, Ca or Mg, but the line showed a significant increase in EC, and a significant decrease in the content of N and S, 2.2 and 1.1 times, respectively [78]. Since most parameters remained unchanged, the observed changes were attributed to the introduction of litter and root exudates into the soil. Another study with virus-resistant papaya, however, obtained different results. It also found no differences in pH and C, but nor did it find any differences in N [78]. The possible causes might have been due to differences in cultivation conditions or plant age (9 months and 9 years), as well as different genotypes used in these two experiments.

Pot cultivation of tobacco with a chitinase gene revealed significant pH fluctuations compared with a control, possibly due to root exudates [81]. Recent field studies did not find any effect on soil physicochemical properties in rice with the *OsCK1* (cholinkinase) gene resistant to rice pyriculariosis and bacterial blight [85], as well as in potato with R-genes resistant to late blight [28].

Transgenic papaya also had a notable effect on soil enzyme activities [78]. A significant increase in activity was observed for alkaline PHO, ARS and invertase, while the protease, polyphenol oxidase and urease activities showed a significant reduction. The activities of acidic PHOs, cellulase, catalase and DHA did not change. The most sensitive enzyme was arylsulfatase, which is involved in the S mineralization in soil; their level grew 5.4 times in the soil of virus-resistant papaya [78]. This was due to the improved growth of the transgenic line; the plant tissues produced more sulfur-containing papain and chymopapain, which required enhanced immobilization of N and S. This, in turn, led to significant differences in the content of N and S, as well as in the ARS activity. Changes in the activities of other enzymes could have been caused by the impact of transgenic papaya on the activity of microorganisms.

The effects of other disease-tolerant transgenic plants on soil biochemistry were insignificant. Field tests of wheat resistant to wheat yellow mosaic virus were conducted for 2 years in two regions of China; cultivation of the GM wheat did not alter the activities of urease, DHA or sucrase [82]. Chitinase-expressing tobacco grown in pots in the chamber house had no effect on protease but showed some effect on catalase, although at the rosette stage only, and on urease, at the stubble stage only [81]. Greenhouse-cultivated oilseed rape plants with a chitinase gene showed no difference from their parent line in C, N, P and S cycle enzymes [84]. Five-year-old white spruce plants (Picea glauca (Moench) Voss), an important commercial species whose wood is used in construction and pulp wood production, were evaluated in the greenhouse [80]. A transformation with the *ech42* gene encoding endochitinase increased the enzyme activity in the spruce roots and root exudates 6 and 2–10 times, respectively, compared to the control; however, the biomass of soil fungi did not change. As has been repeatedly noted in a number of studies, the results of greenhouse assessments of the effects of transgenic trees on soil may differ from those obtained in the field, and therefore long-term field tests are necessary for a conclusive safety assessment of such plants.

### 4.4. Stress-Tolerant Transgenic Plants

Although transgenic plants with tolerance to abiotic stresses are still not common in commercial plantations, their effects on soil have been studied for quite a long time (Table 4). In greenhouse conditions, the roots of transgenic alfalfa plants overexpressing the gene of nodule-enhanced malate dehydrogenase (neMDH)—which confers tolerance to aluminum—produced 7.1 times more organic acids than a control [86]. Subsequent field tests showed a significant increase in the content of P, K, Mn, Cu and Zn in soil from transgenic plants, and a significant decrease in Mg; only the content of Ca and Fe did not change [87]. These observations demonstrate that organic acids produced by plant roots significantly affect the microbial diversity of the rhizosphere and increase the availability of macro- and microelements.

Greenhouse studies did not show any effect of salt-tolerant transgenic plants on the physicochemical properties of soil. These properties (pH, EC, organic C, macro- (N, P, K) and microelements (S, Ca, Mg, Na)), as well as soil texture and density, were not altered by the cultivation of rice with a pea DNA helicase 45 (*PDH45*) gene [89]. No definite trends in soil properties (pH, EC, organic C and N) were observed at any growth stage of two lines of maize with a betaine aldehyde dehydrogenase (*BADH*) gene, either in neutral or in saline–alkaline soil [90]. A 3-year field cultivation of abiotic stress-resistant cotton containing Arabidopsis transcription factor CBF1 did not affect the soil pH, EC, organic matter, P or K [91]. A significant reduction was found only in N, and only in the second year out of three.

Multiple greenhouse studies did not show any effect of stress-tolerant transgenic plants on soil enzyme activities. Two potato lines with a *DREB1A* gene did not alter the activities of urease and β-glucosidase, although some differences were noted in the activities of ARS and alkaline PHOs [87]. Those differences, however, were not confirmed in the second test. Since alkaline PHO is produced only by soil microorganisms [43], changes in its activity are due solely to changes in the microbial activity. These changes could have been caused by fluctuations in temperature and other environmental factors, which could have affected the soil microflora both directly and via changes in plant physiology, e.g., changes in the transgene expression levels [87]. The enzymatic activity of soil was also not changed by the cultivation of various salt-tolerant transgenic species, such as tobacco [88], rice [89] and maize [90].

### 4.5. Transgenic Plants with Modified Metabolic Pathways

An important area in plant genetic engineering is the modification of a crop’s qualitative composition or the content of a certain component. The modifications are most often aimed to improve raw materials for the industry. Generally, a variety of metabolic pathways can be modified in such plants (Table 5), and the resultant new substances or quantitative changes in the existing ones can affect soil microorganisms. For instance, modification of bioenergy crops is used to improve the conversion of lignocellulose biomass into biofuels by manipulating genes of lignin biosynthesis because it is this complex phenolic polymer that prevents access to fermentable polysaccharides [94,95]. Plants with a modified lignin content/composition can affect the soil by altering the uptake of nutrients and/or the composition of plant residues and root exudate [96,97]. Hybrid poplar trees (P. tremula × P. alba) with antisense *CAD* and *COMT* genes for inhibition of lignin biosynthesis were grown in the field for 4 years [98]. The CAD line had significantly lower lignin content, while the COMT lines had a significantly reduced S/G lignin monomer ratio. Yet, the trees showed no effect on total C and N, or MBC in soil. The authors attributed this to a spatial variability of soil properties in the field. Greenhouse cultivation of three tobacco lines with suppressed *CAD* and *COMT* genes of lignin biosynthesis (separately or jointly) did not alter the content of C, soluble carbohydrates or the C/N ratio in the roots, but all the lines had significantly higher MBN [99]. As shown by the measured activities of soil C-cycle enzymes, the cellulase activity in the soil of transgenic plants did not differ from a non-transgenic control, but the roots of the CAD-suppressed line contained significantly more N, and the invertase and xylanase activities were significantly higher in the soil of this line.

The possible effects of modified bioenergy crops on rhizospheric processes, especially those related to C accumulation in soil, were assessed in a field study on two transgenic millet lines (*Panicum virgatum* L.) with downregulation of caffeic acid 3-O-methyltransferase (COMT) [106]. In the first two years, the plants did not affect the pH and concentrations of 19 soil elements. The roots of 5-year-old plants did not differ in lignin content from the control, but, due to a lower content of syringyl (S) monomers, the S/G ratio of the two lines decreased by 40.1% and 42.7% versus the control. However, this did not affect the total SOC content in the upper (0–15 cm) and deeper soil layers (15–30 cm) after 5 years of cultivation [102]. Since changes in SOC occur very slowly, the subsequent study focused on the active fractions of SOC, namely, dissolved organic carbon (DOC) and permanganate oxidizable carbon (POXC), which are more sensitive to changes [107]. In addition to two lines with the suppressed *COMT* gene, the study also used three millet lines overexpressing the PvMYB4 (MYB4) enzyme, the transcription repressor of many lignin biosynthesis genes. After 5–6 years of cultivation, there were no differences in total SOC, DOC and POXC between the COMT and MYB4 lines and the control. Since the aboveground biomass was removed at the end of each growing season, SOC was only dependent on C coming from the roots [107]. The absence of differences in SOC was probably due to the absence of changes in the lignin content in millet roots with suppressed COMT [106]. It should be noted that the aboveground biomass cannot always be removed in production systems; therefore, future studies should include assessments of SOC dynamics at different rates of aboveground biomass removal [107].

Field tests showed that transgenic plants with the modified biosynthesis of less important compounds had an insignificant effect on soil properties. Soybean expressing an Arabidopsis cystathionine-c-synthase gene, which increases the methionine content, had no effect on the total C and N in soil, nor did it differ from a non-transgenic control in the content of amino acids in root exudates [103]. Rice modified to produce resveratrol, a non-flavonoid polyphenol, which is not normally synthesized in cereals due to the absence of resveratrol synthase, had no significant effect on the soil pH, EC, available P, Ca, K, Mg, and Na cations, total N and organic matter [105]. There was also no effect of such plants on the activity of microorganisms. Potato modified to produce amylase-free starch, which may be important for a range of industrial applications, had no effect on laccase, cellulose and Mn-peroxidase [100], or on the microbial catabolic diversity [104]. Potato was also modified to synthesize cyanophycin, a protein polymer used to obtain polyaspartate, which is a biodegradable substitute for the synthetic polymer polyacrylate [101]. Potato tubers left in soil for three winter seasons were shown to have no effect on six enzymes representing the main pathways of the C, N and P cycles in soil.

## 5. Factors Influencing the Interaction of Transgenic Plants with Soil

Most studies have not shown any significant effect of transgenic plants on soil. Some authors reported a number of significant changes but, for the most part, these were inconsistent and transient. The lack of result consistency is probably due to a large variety of interactions among plant parameters (genotype, inserted gene, stage of development) and multiple external factors. These factors include the location, main soil type and climatic conditions, including weather changes during the season and from year to year (temperatures and precipitation), which often explain most observed changes, as well as management systems, which include the use of fertilizers and pesticides, as well as various crop rotation systems [8,15]. All these factors can mask the effects of genetic modification, and it has been repeatedly shown that their influence is greater than that of the transgenic status.

To assess the dynamics of changes in soil, one needs several samples taken during the season, either at different stages of plant development or at certain intervals (which is a less common practice). Rare reports of significant changes in pH, normally a very stable soil parameter, were associated with season. Such findings were reported for pot-cultivated tobacco with a chitinase gene [81] and for field-grown Bt poplars [70]. Supposedly, these changes were caused not by the plants’ transgenic nature but rather by environmental factors. A significant decrease in the content of N in the soil of Bt cotton in the middle of the growing season [58] or that of available P during flowering [63] suggests higher nutrient uptake at certain development phases compared to controls. Changes in TOC, total N and available P depending on the growth stage were also reported for Bt rice [53] and salt-tolerant maize [91]. They could have been caused by, e.g., increased growth.

A recent study by Chen et al. [108] did not investigate a limited number of elements but rather performed a comprehensive profiling of the soil metabolomes of Bt maize and a non-transgenic variety. Soil metabolomics profiling generated a total of 1730 compounds that differed at each of the six growth stages. The degree of changes in metabolites increased up to the fourth stage (heading), and then decreased. This was probably due to changes in plant physiology in transition from vegetative to reproductive growth, which altered the root metabolism. Despite obvious differences in the soil metabolic profiles of the two maize varieties, changes in the rhizosphere bacterial community were associated with the development stages rather than with the genetic modification of plants [108].

A number of authors have reported that within-season changes in soil enzyme activities in Bt plants have little to do with their genetic transformation, but are more likely caused by such factors as differences in the soil water content, temperature fluctuations, use of fertilizers, etc. [55,62,68]. The small magnitude of the effect of transgenic status on soil microbial communities, compared to that of growth stage, was also reported for herbicide-resistant plants [8]. Since plants can alter their exudate composition depending on their development stage, this may be another explanation for changes in soil microbial activity during the season [109].

Long-term tests demonstrated significant effects not only of growth stage but also of year, as was observed for Bt maize [17,66] and Bt cotton [9]. For example, a significant reduction in MBC was observed in maize plants in a year with poorly distributed rainfall [54], while the highest MBC value was in the hottest year in a five-year study [74]. Moreover, not only were the growing season conditions important, but also those of the winter. The activities of enzymes involved in the cycles of C, N and P significantly differed among three winter periods when cyanophycin-producing GM potato tubers were left in soil [105].

Classical breeding varieties are often obtained by crossing various genotypes within, or sometimes between, species, which leads to a large genetic diversity. It is obvious that such varieties can have a significant impact on soil, incomparable with that of a variety obtained by the insertion of one transgene. In most cases, the effect of transgenic modification was found to be insignificant compared to the variability among varieties obtained by traditional breeding. For instance, the effect of Bt maize was within the variation range of the effects of ten conventional varieties [64]. In the study by Khan et al. [84], two oilseed rape varieties significantly differed in the activities of BGL (2.3 times) and ARS (1.5 times), while transgenic plants did not differ from their parents belonging to different varieties.

Fluctuations in the soil content of organic C and macroelements due to the cultivation of transgenic plants could be dependent on the availability of nutrients and water. Field tests with Bt plants showed a significant increase in P under dry land conditions [69] or a significant reduction in NO_3_ and P without the use of fertilizers [32]. Possibly, the differences between transgenic and non-transgenic plants appeared only under stress (drought or nutrient deficiencies) and disappeared under favorable conditions. Small changes in pH at the end of a greenhouse experiment with a transgenic melon [83] were associated with changes in the soil buffer capacity due to added fertilizers. Organic amendments added to the soil were reported to have changed the microbial activity and the bacteria-to-fungi ratio [110]. According to [111], the physicochemical properties of soil, its microbial biomass and activity were sensitive to the introduction of N and P, and the response largely depended on the rate of their application.

Along with the decomposition of organic compounds and nutrient cycling, soil microorganisms are also responsible for the decomposition of pesticides [75]. Therefore, when evaluating GMPs with such traits as resistance to pests, herbicides or diseases, it is also important to take into account the effects of new technologies involving the use of new pesticides or reducing the doses and number of treatments with the old ones. Studies with Bt crops showed no differences among transgenic, control and insecticide-treated control plants in MBC [54], PHO [56], or MBC and BGL [66]. Other studies demonstrated significant differences in MBC [17] or DHA [57], but those differences were not stable during the season or from year to year. The absence of differences between the effects of imazapyr and conventional herbicides on MBC and MBN in the field was confirmed in various soil and climatic conditions covering the main biomes of Brazil [75]. A fungicide treatment of potato plants with late blight resistance also had no effect on the physicochemical properties of soil in 2-year field trials in Ireland and the Netherlands [28].

Large-scale studies, which included the assessment of a number of factors, such as site location, management system and growth stage, demonstrated their greater impact on soil compared with that of an inserted transgene. The effects of glyphosate-resistant plants on soil microorganisms were minor and inconsistent compared to the effects of growing site location and crop rotation [73]. The effect of glyphosate-resistant soybean on soil microbial biomass was insignificant compared to those of site, growing season and soybean cultivar [76]. The potato plant growth stage and field location affected the soil enzyme activities more than the starch modification of tubers [100]. Compared to harvest year, plant growth stage and cotton cultivar, the pest resistance trait had a negligible effect on the activities of DHA, urease and phosphatase in soil [67]. The year and stage of growth significantly affected the urease, DHA and sucrase activities in the field trials of virus-resistant wheat [82].

In hybrid poplar (*Populus alba* × *P. tremula*) expressing a prokaryotic *tzs* gene, the level of cellular cytokinin (trans-zeatin) increased 20-fold, which caused an increase in the aboveground biomass and a number of changes in the plant growth, development and biochemical composition [112]. Field tests of three poplar lines were carried out in three locations in South Korea that differed significantly in pH, NPK content and soil texture [113]. A significant difference in microbial biomass was found in one of the three locations and in one clone. The change was possibly caused by the genetic transformation (changes in the composition of exudates), but it was temporary and associated with location and genotype.

## 6. Effects of Transgenic Plant Residues

Decomposition of plant residues is a key function in element cycling, and any change in their composition can affect the functions of soil [15]. There were reports of unintended effects such as a 33% to 97% increase in the lignin content in maize [114], changes in the C and N content [66,104], and increases in the underground [65] and aboveground [53] biomass. Most studies of litter decomposition have assessed the decomposition rate by measuring weight loss and C emission, and only a few assessed the effects on microorganisms. In the study by Wu et al. [115], PHO was insensitive to the decomposition of Bt rice straw, which contained significantly more N, P, K and unchanged C. At the same time, the activity of DHA sharply increased in the initial phase of the experiment (the first two weeks out of 12). The higher content of macronutrients in the Bt straw possibly contributed to the significant growth of the microbial population, but the effect was temporary. The effect instability was also noted in [116], where they studied the decomposition of salt-resistant maize straw in neutral or in saline–alkali soil for 7 months. The transgenic straw did not differ from the control in cellulose and lignin, but its C/N ratio was lower. The early stage of its decomposition was faster in the saline–alkali soil and this significantly increased MBC and MBN; by the end of the experiment, however, there were no differences in microbial biomass between the transgenic straw and control. Potato tubers with modified starch composition did not differ in the content of lignin, cellulose and non-cellulose polysaccharides [117]. When they were left to decompose in litterbags in the growing soil, there were significant differences from the control in the activities of laccase, Mn-peroxidase and cellulose. It is not clear, however, if the enzymes in the residue sphere were produced there or were leaching out of the bags.

Of particular interest is understanding how soil processes are affected by litter from transgenic trees that grow in one place for a long time. Stems and roots with a modified lignin composition were shown to initially decompose faster due to a lower protection of labile plant components from enzymatic attack [94,118]. Trunk segments of 4-year-old poplars with suppression of lignin biosynthesis genes *CAD* and *COMT* were left to decompose in soil from three different locations [119]. After 552 days of the experiment, the adhering layer of soil (detritisphere) was analyzed for MBN. While the original wood samples did not differ in C and N, there was a significant difference in soil MBN by the end of the experiment. However, the effects of the genetic transformation on MBN were not consistent across different soils. Fungi are known to have a more important part to play than bacteria in litter decomposition [120]. A study by Vauramo et al. [121] determined the fungal biomass in decomposing leaves from birch with an antifungal chitinase gene. The C/N ratio in the transgenic leaves did not differ from the control, and the 11-month experiment failed to find any effect on the fungal biomass.

In hot climate countries, plant residues can be used as mulch to preserve moisture in the soil, control weeds and improve nutrient availability [122]. The effects of Bt cotton mulch on soil properties (pH, EC, macro- and microelements), weed growth and productivity of winter crops—wheat, Egyptian clover and canola—were studied in Pakistan [123]. Toxins released by Bt mulch reduced weed density, but negatively affected the winter crop productivity and did not significantly affect the properties of soil. This should be taken into account when selecting crops for rotation.

## 7. Future Prospects

To date, the effects on soil processes have been studied for GMPs with various traits (Table 1, Table 2, Table 3, Table 4 and Table 5). A large number of greenhouse and field studies were carried out to identify possible deviations from non-transgenic plants, which also assessed a number of factors related to both the plant (growth stage, genotype features) and growing conditions (site location, changes in management systems). In most reports, the detected changes were within the limits of statistical error, and in the case of statistical significance, they were most often temporary and were not reproduced at the next sampling in the season or the next year. No specific effect of a particular type of transgenic plant (e.g., pest or herbicide-resistant) has been identified, and the lack of generally accepted experimental design and evaluation criteria makes such comparison difficult. For example, some researchers noted the effect of Bt plants on the content of available P, but this could also be caused by increased growth, and biomass was not always measured. In general, no unequivocally negative or positive effect of any transgenic genotype on the physicochemical or microbial properties of the soil has been shown. However, some groups of transgenic plants or their possible effects have not been given due attention, and research in these areas can be expanded.

The development of transgenic plants with increased productivity by improving the nutrient use efficiency or photosynthesis is a popular area in plant genetic engineering. A wide range of genes and approaches have been applied to improve the plant use efficiency of N [124,125], P [126] and other nutrients [127,128], as well as to optimize photosynthesis [129,130]. Such plants would of course need more nutrients for increased biomass production, yet their impact on soil processes has barely been studied and further research is needed. Our group studied the effects of transgenic birch and aspen plants with the pine glutamine synthetase *GS1* gene on the physicochemical properties, enzyme activity and microbial biomass of soil. Four-year pot experiments showed that transgenic plants differed in growth rate and C and N content, but differences in enzyme activity and microbial biomass were temporary and inconsistent. However, by the end of the experiments, we observed a decrease in soil K, possibly due to its enhanced uptake to neutralize secondary NH4 reassimilated via glutamine synthetase (unpublished data).

In addition to common substances such as carbohydrates, proteins, organic acids and amino acids, root exudates of some plants may contain allelochemicals involved in rhizospheric interactions between plants and other organisms [131]. These substances can also affect soil microorganisms. The neem tree (*Azadirachta indica* Juss) from tropical Asia contains a number of allelochemicals, the most toxic of which is the alkaloid azadirachtin, which has insecticidal activity [132]. When added into soil, azadirachtin granules did not affect DHA but significantly altered the PHO activity: increased at the recommended dose (1×) and inhibited at five times (5×) the recommended dose [133]. For environmentally safe weed control, it was proposed to use allelopathic rice capable of inhibiting the growth of neighboring plants [134]. This is achieved owing to root exudates containing allelochemicals, the most important of which is flavone O-glycoside. In different growth phases, the effect of allelopathic rice on soil enzymes (DHA, polyphenol oxidase, urease and invertase) was positive or neutral, although the flavone O-glycoside concentration in soil was always about five times that in control. The potential mechanism of this effect remains unclear [134].

The influence of genetic transformation on changes in the composition and/or content of allelochemicals is still poorly understood. This issue takes on particular importance with regard to artificial tree plantations, where a prolonged cultivation and the dominance of one species can lead to the accumulation of allelochemicals to toxic levels [135]. Allelopathic tree species include, among others, some important fruit (walnut) and forest (eucalypts, some coniferous species) species [136]. Eucalypt species are currently the most important in the plantation forestry. They are widely used for genetic transformation, and a fast growing eucalypt was approved for commercial use in Brazil in 2015 [137]. Most eucalypt species are known to have an allelopathic influence in nature [138]. To date, allelopathy tests for environment biosafety have been conducted on salt-tolerant eucalypts overexpressing various genes [138,139,140] and on eucalypt with reduced lignin content [141]. The tests assessed lettuce seed germination on agar with added dried leaves (sandwich method) or in soil used in growing transgenic eucalypts. The abundance of microorganisms and their function were not studied, so further research should be conducted.

Long-term cultivation of GMPs, particularly the woody ones, in one place can affect not only the very labile microbiological parameters (enzyme activity, microbial biomass), but also such lengthy processes as the decomposition of plant residues, mineralization of organic matter and accumulation of soil carbon. Unlike the case with annual crops, both root exudates and residues of trees can accumulate in soil, and even minor changes can build up over time. However, long-term field tests are quite expensive and time-consuming and they still cannot take account of all possible soil and climatic conditions in which the plants can be grown, as well as various applicable management systems. Meanwhile, long-term effects on soil can be assessed using a mathematical modeling of various scenarios. For example, modeling of the 30- and 60-year cultivation of transgenic aspen plantations with a modified wood composition under Northern Eurasia conditions showed 5–7% changes in soil C and N pools, which do not exceed the effects of a standard silvicultural management [142].

Unfortunately, there have been virtually no studies that have simultaneously assessed the effects of transgenic plants on the quantitative (microbial biomass), functional (soil enzymes) and qualitative (diversity) characteristics of microbial communities. Conducting such studies would help towards a better understanding of the relationships between the diversity and functions of microorganisms in soil used to cultivate transgenic plants.

## Figures and Tables

**Table 1:** Risk assessment of insect-resistant transgenic plants on soil quality.

Species	Gene	Growth Conditions	Indicators	Additional Factors	References
maize	*Cry3Bb*	field (2 years)	MBC ^1^	growth stage	[54]
				insecticide	
maize	*Cry3Bb*	field (3 years)	MBC	growth stage	[17]
				insecticide	
maize	*Cry1Ab, Cry3Bb1*	field (4 years)	N, P(2), S, DHA ^2^	variety	[55]
rice	*Cry1Ab*	field (2 years)	P, DHA	growth stage	[56]
				insecticide	
maize	*Cry1Ab*	field	N, DHA	growth stage	[57]
cotton	*Cry1Ac*	net house	NH4, NO3, N, P ^3^	growth stage	[58]
			DHA		
cotton	*Cry1Ac*	net house	organic C	growth stage	[59]
			N, P(2)		
			MBC, MBN, MBP		
maize	*Cry1Ab*	field (7 years)	C, N, texture		[60]
cotton	*Cry1Ac*	field (3 years)	N(2), P, DHA		[61]
cotton	*Cry1Ac*	greenhouse (4 years)	C, N(3), P(2), S, DHA, CAT		[9]
	*Cry1Ac* + *CpTI*		MBC		
rice	*Cry1Ac*	open air (pots)	C(2), N(2), P, DHA	growth stage	[62]
cotton	*Cry1Ac*	pots	org. matter, N, P, K	growth stage	[63]
			C, N(2), P, DHA		
maize	*Cry1Ab*	climate chamber	DHA	variety	[64]
			MBC, MBN		
maize	*Cry1Ab*	climate chamber	DHA	soil type	[65]
			MBC, MBN		
maize	*Cry1Ab*	field (5 years)	C	insecticide	[66]
			MBC	crop rotation	
cotton	*cry1Ac*	field	N, DHA		[40]
			MBC		
cotton	*CrylAc* + *CpTI*	field (2 years)	N, P, DHA	growth stage	[67]
sugarcane	*Cry1Ac*	field	C, N(2), P	growth stage	[68]
cotton	*Cry1Ac*	field	pH, EC, org. C, NO3, NH4, P, K	fertilization	[32]
			MBC		
cotton	*Cry1Ac*	field (6 years)	C, N(3), P(2), S, DHA		[10]
			MBC		
rice	*Cry1Ac*	field (8 years)	org. C, N, P, C/N	growth stage	[53]
			P, N, DHA, CAT		
			MBC, MBN		
cotton	*Cry1Ac*	field (2 years)	N, P(2), CAT	growth stage	[12]
			MBC	salinity	
maize	*Cry1Ab*	field	pH, org. C, NO3, NH4, P	irrigation	[69]
			C, N, P		
poplar	*Cry1Ac, Cry3A*	field (5 years)	pH, org. matter, N, P, K	growth stage	[70]
cotton	*Bt*	field	N, P, K	crop rotation	[71]
			P, DHA		
			MBC		
poplar	*Cry1Ah1*	field (4 years)	pH, N, P		[11]
			MBC, MBN, MBP		

^1^ Microbial biomass (MBC, MBN, MBP). ^2^ Enzyme activity: element cycle (enzyme number). ^3^ Physicochemical soil properties.

**Table 3:** Risk assessment of disease-tolerant transgenic plants on soil quality.

Species	Gene	Growth Conditions	Indicators	Additional Factors	References
papaya	*PRSV CP*	field	pH, org. C, N		[79]
papaya	*PRSV RP*	open air (pots)	pH, EC, org. matter, N, P, K, microel.		[78]
			C(3), N(2), P(3), S, DHA, CAT		
spruce	*ech42*	greenh. (5 years)	fungal biomass		[80]
tobacco	*McChit1*	chamber house	pH	growth stage	[81]
			N(2), CAT		
wheat	*WYMV-Nib8*	field (2 years)	C, N, DHA	growth stage	[82]
				location	
melon	*AFP* + *CHI*	greenhouse	pH, EC, org. matter, P, K		[83]
			C(2), N(2), P(2), S, DHA, CAT		
oilseed rape	*NiC*	greenhouse	C (2), N, P, S	variety	[84]
rice	*OsCK1*	field	pH, EC, org. matter, N, P, K, microel.	growth stage	[85]
potato	*Rpi-vnt1.1*	field (2 years)	pH, org. C, N, C/N	variety	[28]
				fungicide	
				location	

**Table 4:** Risk assessment of stress-tolerant transgenic plants on soil quality.

Species	Gene	Growth Conditions	Indicators	Additional Factors	References
alfalfa	*MDH*	field	pH, P, K, micro		[87]
potato	*DREB1A*	greenhouse	C, N, P, S	salinity	[88]
tobacco	*MCM6*	greenhouse	P, DHA	salinity	[89]
rice	*PDH45*	greenhouse	pH, EC, org. C, N, P, K, microel., texture	salinity	[90]
			N(2), P, DHA	soil type	
maize	*BADH*	greenhouse	pH, EC, org. C, N	growth stage	[91]
			C, N, CAT	soil type	
cotton	*CBF1*	field (3 years)	pH, EC, org. matter, N, P, K		[92]
maize	*BADH*	field (3 years)	C, N, DHA	growth stage	[93]

**Table 5:** Risk assessment of metabolic engineered transgenic plants on soil quality.

Species	Gene	Growth Conditions	Indicators	Additional Factors	References
poplar	*CAD, COMT* (AS) ^1^	field (4 years)	C, N	location	[98]
			MBC		
potato	*GBSS* (RNAi)	field (3 years)	C(2), PER	growth stage	[100]
				variety	
				location	
potato	*cphA*	field (3 years)	C(4), N(2), P	growth stage	[101]
rice	*pacrtB* + *pacrtE* +	field	C, N(2), CAT	growth stage	[102]
	*pacrtY* + *pacrtI*				
soybean	*AtD-CGS*	field	C, N		[103]
tobacco	*CAD, COMT, CCR*	greenhouse	MBN		[99]
			C(3)		
potato	*GBSS* (RNAi) ^2^	field	org. matter	growth stage	[104]
				location	
rice	*AhSTS1*	field	pH, EC, org. matter, N, P, K, microel.	growth stage	[105]
switchgrass	*COMT* (RNAi)	field (5 years)	pH, org. matter, P, K, microel.		[106]
switchgrass	*COMT* (RNAi)	field (5–6 years)	organic C		[107]
	*MYB*				

^1^ AS = antisense. ^2^ RNAi = RNA interference.

## Data Availability

Not applicable.

## References

[1] ISAAA (2019). Global Status of Commercialized Biotech/GM Crops in 2019: Biotech Crops Drive Socio-Economic Development and Sustainable Environment in the New Frontier.

[2] Snow A.A., Palma P.M. (1997). Commercialization of transgenic plants: Potential ecological risks. BioScience.

[3] Messeguer J. (2003). Gene flow assessment in transgenic plants. Plant Cell Tissue Organ Cult..

[4] Sisterson M.S., Carrière Y., Dennehy T.J., Tabashnik B.E. (2006). Evolution of resistance to transgenic crops: Interactions between insect movement and field distribution. J. Econ. Entomol..

[5] Romeis J., Meissle M., Bigler F. (2006). Transgenic crops expressing Bacillus thuringiensis toxins and biological control. Nat. Biotechnol..

[6] Bruinsma M., Kowalchuk G.A., van Veen J.A. (2003). Effects of genetically modified plants on microbial communities and processes in soil. Biol. Fertil. Soils.

[7] Singh A.K., Dubey S.K., Dubey S.K., Pandey A., Sangwan R.S. (2017). 8—Transgenic Plants and Soil Microbes. Current Developments in Biotechnology and Bioengineering.

[8] Guan Z., Lu S., Huo Y., Guan Z.-P., Liu B., Wei W. (2016). Do genetically modified plants affect adversely on soil microbial communities?. Agric Ecosyst. Environ..

[9] Chen Z., Chen L., Wu Z. (2012). Relationships among persistence of Bacillus thuringiensis and Cowpea trypsin inhibitor proteins, microbial properties and enzymatic activities in rhizosphere soil after repeated cultivation with transgenic cotton. Appl. Soil Ecol..

[10] Chen Z., Wei K., Chen L., Wu Z., Luo J., Cui J. (2017). Effects of the consecutive cultivation and periodic residue incorporation of Bacillus thuringiensis (Bt) cotton on soil microbe-mediated enzymatic properties. Agric Ecosyst. Environ..

[11] Wei H.W., Movahedi A., Liu G., Kiani-Pouya A., Rasouli F., Yu C., Chen Y., Zhong F., Zhang J. (2022). Effects of field-grown transgenic Cry1Ah1 poplar on the rhizosphere microbiome. Res. Sq..

[12] Luo J.-Y., Zhang S., Zhu X.-Z., Lu L.-M., Wang C.-Y., Li C.-H., Cui J.-J., Zhou Z.-G. (2017). Effects of soil salinity on rhizosphere soil microbes in transgenic Bt cotton fields. J. Integr. Agric..

[13] Haichar F.Z., Marol C., Berge O., Rangel-Castro J.I., Prosser J.I., Balesdent J., Achouak W. (2008). Plant host habitat and root exudates shape soil bacterial community structure. ISME J..

[14] Guyonnet J.P., Cantarel A.A.M., Simon L., Haichar F.Z. (2018). Root exudation rate as functional trait involved in plant nutrient-use strategy classification. Ecol. Evol..

[15] Hannula S.E., de Boer W., van Veen J.A. (2014). Do genetic modifications in crops affect soil fungi?. A review. Biol. Fertil. Soils.

[16] Mandal A., Sarkar B., Owens G., Thakur J.K., Manna M.C., Niazi N.K., Jayaraman S., Patra A.K. (2020). Impact of genetically modified crops on rhizosphere microorganisms and processes: A review focusing on Bt cotton. Appl. Soil Ecol..

[17] Devare M., Londono-R L.M., Thies J.E. (2007). Neither transgenic Bt maize (MON863) nor tefluthrin insecticide adversely affect soil microbial activity or biomass: A 3-year field analysis. Soil Biol. Biochem..

[18] Dunfield K.E., Germida J.J. (2004). Impact of genetically modified crops on soil- and plant-associated microbial communities. J. Environ. Qual..

[19] Larson W.E., Pierce F.J. (1991). Conservation and enhancement of soil quality. Evaluation for Sustainable Land Management in the Developing World.

[20] Beule L., Vaupel A., Moran-Rodas V.E. (2022). Abundance, diversity, and function of soil microorganisms in temperate alley-cropping agroforestry systems: A review. Microorganisms.

[21] McGill B.J., Enquist B.J., Weiher E., Westoby M. (2006). Rebuilding community ecology from functional traits. Trends Ecol. Evol..

[22] Singh B.K., Quince C., Macdonald C.A., Khachane A., Thomas N., Al-Soud W.A., Sørensen S.J., He Z., White D., Sinclair A. (2014). Loss of microbial diversity in soils is coincident with reductions in some specialized functions. Environ. Microbiol..

[23] Escalas A., Hale L., Voordeckers J.W., Yang Y., Firestone M.K., Alvarez-Cohen L., Zhou J. (2019). Microbial functional diversity: From concepts to applications. Ecol. Evol..

[24] Peter H., Beier S., Bertilsson S., Lindstrom E.S., Langenheder S., Tranvik L.J. (2011). Function-specific response to depletion of microbial diversity. ISME J..

[25] Plante C.J. (2017). Defining disturbance for microbial ecology. Microb. Ecol..

[26] Chen H., Ma K., Huang Y., Yao Z., Chu C. (2021). Stable soil microbial functional structure responding to biodiversity loss based on metagenomic evidences. Front. Microbiol..

[27] Louca S., Jacques S.M.S., Pires A.P.F., Leal J.S., Srivastava D.S., Parfrey L.W., Farjalla V.F., Doebeli M. (2016). High taxonomic variability despite stable functional structure across microbial communities. Nat. Ecol. Evol..

[28] Krause S.M.B., Näther A., Cortes V.O., Mullins E., Kessel G.J.T., Lotz L.A.P., Tebbe C.C. (2020). No tangible effects of field-grown cisgenic potatoes on soil microbial communities. Front. Bioeng. Biotechnol..

[29] Lauber C.L., Hamady M., Knight R., Fierer N. (2009). Pyrosequencing-based assessment of soil pH as a predictor of soil bacterial community structure at the continental scale. Appl. Environ. Microbiol..

[30] Lee Y.H., Ahn B.K., Sonn Y.K. (2011). Effects of electrical conductivity on the soil microbial community in a controlled horticultural land for strawberry cultivation. Korean J. Soil Sci. Fert..

[31] Zhang C., Liu G., Xue S., Song Z. (2011). Rhizosphere soil microbial activity under different vegetation types on the Loess Plateau, China. Geoderma.

[32] Ahamd M., Abbasi W.M., Jamil M., Iqbal M., Hussain A., Akhtar M.F., Nazli F. (2017). Comparison of rhizosphere properties as affected by different Bt- and non-Bt-cotton (Gossypium hirsutum L.) genotypes and fertilization. Environ. Monit. Assess..

[33] Song Y., Song C., Yang G., Miao Y., Wang J., Guo Y. (2012). Changes in labile organic carbon fractions and soil enzyme activities after marshland reclamation and restoration in the Sanjiang Plain in Northeast China. Environ. Manag..

[34] Hinojosa M.B., Carreira J.A., Garcıa-Ruız R., Dick R.P. (2004). Soil moisture pre-treatment effects on enzyme activities as indicators of heavy metal-contaminated and reclaimed soils. Soil Biol. Biochem..

[35] Lino I.A.N., Santos V.M., Escobar I.E.C., Silva D.K.A., Maia L.C. (2016). Soil enzymatic activity in Eucalyptus grandis plantations of different ages. Land Degrad. Dev..

[36] Brzezińska M., Lipiec J., Frąc M., Oszust K., Szarlip P., Turski M. (2018). Quantitative interactions between total and specific enzyme activities and C and N contents in earthworm-affected pear orchard soil. Land Degrad. Devel..

[37] Gil-Sotres F., Trasar-Cepeda C., Leiros M.C., Seoane S. (2005). Different approaches to evaluating soil quality using biochemical properties. Soil Biol. Biochem..

[38] Garcia C., Hernandez T., Costa F. (1997). Potential use of dehydrogenase activity as an index of microbial activity in degraded soils. Commun. Soil Sci. Plant Anal..

[39] Eivazi F., Tabatabai M.A. (1988). Glucosidases and galactosidases in soils. Soil Biol. Biochem..

[40] Velmourougane K., Sahu A. (2013). Impact of transgenic cottons expressing cry1Ac on soil biological attributes. Plant Soil Environ..

[41] Jan M.T., Roberts P., Tonheim S.K., Jones D.L. (2009). Protein breakdown represents a major bottleneck in nitrogen cycling in grassland soils. Soil Biol. Biochem..

[42] Kramer S., Green D.M. (2000). Acid and alkaline phosphatase dynamics and their relationship to soil microclimate in a semiarid woodland. Soil Biol. Biochem..

[43] Tarafdar J.C., Claassen N. (1988). Organic phosphorus compounds as a phosphorus source for higher plants through the activity of phosphatase produced by plant roots and microorganisms. Biol. Fertil. Soils.

[44] Kertesz M.A., Mirleau P. (2004). The role of soil microbes in plant sulphur nutrition. J. Exp. Bot..

[45] Frankenberger W.T., Johanson J.B. (1983). Method of measuring invertase activity in soils. PIant Soil.

[46] Gander L.K., Hendricks C.W., Doyle J.D. (1994). Interferences, limitations and an improvement in the extraction and assessment of cellulase activity in soil. Soil Biol. Biochem..

[47] Floch C., Alarcon-Gutiérrez E., Criquet S. (2007). ABTS assay of phenol oxidase activity in soil. J. Microbiol. Methods.

[48] Trasar-Cepeda C., Camina F., Leirós C., Gil-Sotres F. (1999). An improved method to measure catalase activity in soils. Soil Biol. Biochem..

[49] Singh J.S., Raghubanshi A.S., Singh R.S., Srivastava S.C. (1989). Microbial biomass acts as a source of plant nutrients in dry tropical forest and savanna. Nature.

[50] Sparling G.P. (1992). Ratio of microbial biomass carbon to soil organic carbon as a sensitive indicator of changes in soil organic matter. Aust. J. Soil Res..

[51] Chen H., Zhao X., Chen X., Lin Q., Li G. (2018). Seasonal changes of soil microbial C, N, P and associated nutrient dynamics in a semiarid grassland of north China. Appl. Soil Ecol..

[52] Geisseler D., Horwath W.R. (2009). Short-term dynamics of soil carbon, microbial biomass, and soil enzyme activities as compared to longer-term effects of tillage in irrigated row crops. Biol. Fertil. Soils.

[53] Lee Z.L., Bu N.S., Cui J., Chen X.P., Xiao M.Q., Wang F., Song Z.P., Fang C.M. (2017). Effects of long-term cultivation of transgenic Bt rice (Kefeng-6) on soil microbial functioning and C cycling. Sci. Rep..

[54] Devare M.H., Jones C.M., Thies J.E. (2004). Effect of Cry3Bb transgenic corn and tefluthrin on the soil microbial community: Biomass, activity, and diversity. J. Environ. Qual..

[55] Icoz I., Saxena D., Andow D.A., Zwahlen C., Stotzky G. (2008). Microbial populations and enzyme activities in soil in situ under transgenic corn expressing cry proteins from Bacillus thuringiensis. J. Environ. Qual..

[56] Liu W., Lu H.H., Wu W.X., Wei Q.K., Chen Y.X., Thies J.E. (2008). Transgenic Bt rice does not affect enzyme activities and microbial composition in the rhizosphere during crop development. Soil Biol. Biochem..

[57] Oliveira A.P., Pampulha M.E., Bennett J.P. (2008). A two-year field study with transgenic Bacillus thuringiensis maize: Effects on soil microorganisms. Sci. Total Environ..

[58] Sarkar B., Patra A.K., Purakayastha T.J. (2008). Transgenic Bt-cotton affects enzyme activity and nutrient availability in a sub-tropical inceptisol. J. Agron. Crop Sci..

[59] Sarkar B., Patra A.K., Purakayastha T., Megharaj M. (2009). Assessment of biological and biochemical indicators in soil under transgenic Bt and non-Bt cotton crop in a sub-tropical environment. Environ. Monit. Assess..

[60] Kravchenko A.N., Hao X., Robertson G.P. (2009). Seven years of continuously planted Bt corn did not affect mineralizable and total soil C and total N in surface soil. Plant Soil.

[61] Mina U., Chaudhary A., Kamra A. (2011). Effect of Bt cotton on enzymes activity and microorganisms in rhizosphere. J. Agric. Sci..

[62] Wei M., Tan F., Zhu H., Cheng K., Wu X., Wang J., Zhao K., Tang X. (2012). Impact of Bt-transgenic rice (SHK601) on soil ecosystems in the rhizosphere during crop development. Plant Soil Environ..

[63] Yang W., Zhang M., Ding G. (2012). Effect of transgenic Bt cotton on bioactivities and nutrients in rhizosphere soil. Commun. Soil Sci. Plant Anal..

[64] Fließbach A., Messmer M., Nietlispach B., Infante V., Mäder P. (2012). Effects of conventionally bred and Bacillus thuringiensis (Bt) maize varieties on soil microbial biomass and activity. Biol. Fertil. Soils.

[65] Fließbach A., Nietlispach B., Messmer M., Rodriguez-Romero A.-S., Maeder P. (2013). Microbial response of soils with organic and conventional management history to the cultivation of Bacillus thuringiensis (Bt)-maize under climate chamber conditions. Biol. Fertil. Soils.

[66] Lupwayi N.Z., Blackshaw R.E. (2013). Soil microbial properties in Bt (Bacillus thuringiensis) corn cropping systems. Appl. Soil Ecol..

[67] Zhang Y.N., Xie M., Li C.Y., Wu G., Peng D.L. (2014). Impacts of the transgenic CrylAc and CpTI insect-resistant cotton SGK321 on selected soil enzyme activities in the rhizosphere. Plant Soil Environ..

[68] Zhou D., Xu L., Gao S., Guo J., Luo J., You Q., Que Y. (2016). Cry1Ac transgenic sugarcane does not affect the diversity of microbial communities and has no significant effect on enzyme activities in rhizosphere soil within one crop season. Front. Plant Sci..

[69] van Wyk D.A.B., Adeleke R., Rhode O.H.J., Bezuidenhout C.C., Mienie C. (2017). Ecological guild and enzyme activities of rhizosphere soil microbial communities associated with Bt-maize cultivation under field conditions in North West Province of South Africa. J. Basic Microbiol..

[70] Zuo L.H., Yang R.L., Zhen Z.X., Liu J.X., Huang L.S., Yang M.S. (2018). A 5-year field study showed no apparent effect of the Bt transgenic 741 poplar on the arthropod community and soil bacterial diversity. Sci. Rep..

[71] Mandal A., Thakur J.K., Sahu A., Manna M.C., Rao A.S., Sarkar B., Patra A.K. (2019). Effects of Bt-cotton on biological properties of Vertisols in central India. Arch. Agron. Soil Sci..

[72] Sessitsch A., Gyamfi S., Tscherko D., Gerzabek M.H., Kandeler E. (2005). Activity of microorganisms in the rhizosphere of herbicide treated and untreater transgenic glufosinate-tolerant and wildtype oilseed rape grown in containment. Plant Soil.

[73] Lupwayi N.Z., Hanson K.G., Harker K.N., Clayton G.W., Blackshaw R.E., Donovan J.T., Johnson E.N., Gan Y., Irvine R.B., Monreal M.A. (2007). Soil microbial biomass, functional diversity and enzyme activity in glyphosate-resistant wheat–canola rotations under low-disturbance direct seeding and conventional tillage. Soil Biol. Biochem..

[74] Lupwayi N.Z., Blackshaw R.E. (2012). Soil microbiology in glyphosate-resistant corn cropping systems. Agron. J..

[75] Souza R.A., Babujia L.C., Silva A.P., Guimarães M.F., Arias C.A., Hungria M. (2013). Impact of the ahas transgene and of herbicides associated with the soybean crop on soil microbial community. Transgenic Res..

[76] Babujia L.C., Silva A.P., Nakatani A.S., Cantao M.E., Vasconcelos A.T.R., Visentainer J.V., Hungria M. (2016). Impact of long-term cropping of glyphosate-resistant transgenic soybean [Glycine max (L.) Merr.] on soil microbiome. Transgenic Res..

[77] Nakatani A.S., Fernandes M.F., Souza R.A., Silva A.P., Reis-Junior F.B., Mendes I.C., Hungria M. (2014). Effects of the glyphosate-resistance gene and of herbicides applied to the soybean crop on soil microbial biomass and enzymes. Field Crops Res..

[78] Wei X.D., Zou H.L., Chu L.M., Liao B., Ye C.M., Lan C.Y. (2006). Field released transgenic papaya affects microbial communities and enzyme activities in soil. Plant Soil.

[79] Hsieh Y.-T., Pan T.-M. (2006). Influence of planting papaya ringspot virus resistant transgenic papaya on soil microbial biodiversity. J. Agric. Food Chem..

[80] Stefani F.O.P., Tanguay P., Pelletier G., Piche Y., Hamelin R.C. (2010). Impact of endochitinase-transformed white spruce on soil fungal biomass and ectendomycorrhizal symbiosis. Appl. Environ. Microbiol..

[81] Wang B., Shen H., Yang X., Guo T., Zhang B., Yan W. (2013). Effects of chitinase-transgenic (McChit1) tobacco on the rhizospheric microflora and enzyme activities of the purple soil. Plant Soil Environ..

[82] Wu J., Yu M., Xu J., Du J., Ji F., Dong F., Li X., Shi J. (2014). Impact of transgenic wheat with wheat yellow mosaic virus resistance on microbial community diversity and enzyme activity in rhizosphere soil. PLoS ONE.

[83] Bezirganoglu I., Uysal P. (2017). Impact of transgenic AFPCHI (Cucumis melo L. Silver Light) fungal resistance melon on soil microbial communities and enzyme activities. J. Plant Biotechnol..

[84] Khan M.S., Sadat S.U., Jan A., Munir I. (2017). Impact of transgenic Brassica napus harboring the antifungal synthetic chitinase (NiC) gene on rhizosphere microbial diversity and enzyme activities. Front. Plant Sci..

[85] Sohn S.-I., Oh Y.-J., Ahn J.-H., Kang H.-J., Cho W.-S., Cho Y., Lee B.K. (2019). Effects of disease resistant genetically modified rice on soil microbial community structure according to growth stage. Korean J. Environ. Agric..

[86] Tesfaye M., Temple S.J., Allan D.L., Vance C.P., Samac D.A. (2001). Over-expression of malate dehydrogenase in transgenic alfalfa enhances organic acid synthesis and confers tolerance to aluminum. Plant Physiol..

[87] Tesfaye M., Dufault N.S., Dornbusch M.R., Deborah L., Allan D.L., Vance C.P., Samac D.A. (2003). Influence of enhanced malate dehydrogenase expression by alfalfa on diversity of rhizobacteria and soil nutrient availability. Soil Biol. Biochem..

[88] Mimura M., Lelmen K.E., Shimazaki T., Kikuchi A., Watanabe K.N. (2008). Impact of environmental stress-tolerant transgenic potato on genotypic diversity of microbial communities and soil enzyme activities under stress conditions. Microbes Environ..

[89] Chaudhry V., Dang H.Q., Tran N.Q., Mishra A., Chauhan P.S., Gill S.S., Nautiyal C.S., Tuteja N. (2012). Impact of salinity-tolerant MCM6 transgenic tobacco on soil enzymatic activities and the functional diversity of rhizosphere microbial communities. Res. Microbiol..

[90] Sahoo R.K., Tuteja N. (2013). Effect of salinity tolerant PDH45 transgenic rice on physicochemical properties, enzymatic activities and microbial communities of rhizosphere soils. Plant Signal. Behav..

[91] Bai X., Zeng X., Huang S., Liang J., Dong L., Wei Y., Li Y., Qu J., Wang Z. (2019). Marginal impact of cropping BADH transgenic maize BZ-136 on chemical property, enzyme activity, and bacterial community diversity of rhizosphere soil. Plant Soil.

[92] Tian W.-H., Yi X.-L., Liu S.-S., Zhou C., Wang A.-Y. (2020). Effect of transgenic cotton continuous cropping on soil bacterial community. Ann. Microbiol..

[93] Zeng X., Pei T., Song Y., Guo P., Zhang H., Li X., Li H., Di H., Wang Z.A. (2022). Three-year plant study of salt-tolerant transgenic maize showed no effects on soil enzyme activity and nematode community. Life.

[94] Li Q., Song J., Peng S., Wang J.P., Qu G.Z., Sederoff R.R., Chiang V.L. (2014). Plant biotechnology for lignocellulosic biofuel production. Plant Biotechnol. J..

[95] Lebedev V.G., Shestibratov K.A. (2021). Genetic engineering of lignin biosynthesis in trees: Compromise between wood properties and plant viability. Russ. J. Plant Physiol..

[96] Motavalli P.P., Kremer R.J., Fang M., Means N.E. (2004). Impact of genetically modified crops and their management on soil microbially mediated plant nutrient transformations. J. Environ. Qual..

[97] Kolseth A.-K., D’Hertefeldt T., Emmerich M., Forabosco F., Marklund S., Cheeke T.E., Hallin S., Weih M. (2015). Influence of genetically modified organisms on agro-ecosystem processes. Agric. Ecosyst. Environ..

[98] Pilate G., Guiney E., Holt K., Petit-Conil M., Lapierre C., Leplé J.-C., Pollet B., Mila I., Webster. E.A., Marstorp H.G. (2002). Field and pulping performances of transgenic trees with altered lignification. Nat. Biotechnol..

[99] Tilston E.L., Halpin C., Hopkins D.W. (2014). Simultaneous down-regulation of enzymes in the phenylpropanoid pathway of plants has aggregated effects on rhizosphere microbial communities. Biol. Fertil. Soils.

[100] Hannula S.E., de Boer W., van Veen J. (2012). A 3-Year study reveals that plant growth stage, season and field site affect soil fungal communities while cultivar and GM-trait have minor effects. PLoS ONE.

[101] Lahl K., Unger C., Emmerling C., Broer I., Thiele-Bruhn S. (2012). Response of soil microorganisms and enzyme activities on the decomposition of transgenic cyanophycin-producing potatoes during overwintering in soil. Eur. J. Soil Biol..

[102] Li P., Dong J., Yang S., Bai L., Wang J., Wu G., Wu X., Yao Q., Tang X. (2014). Impact of β-carotene transgenic rice with four synthetic genes on rhizosphere enzyme activities and bacterial communities at different growth stages. Eur. J. Soil Biol..

[103] Liang J., Sun S., Ji J., Wu H., Meng F., Zhang M., Zheng X., Wu C., Zhang Z. (2014). Comparison of the rhizosphere bacterial communities of zigongdongdou soybean and a high-methionine transgenic line of this cultivar. PLoS ONE.

[104] Brolsma K.M., Vonk J.A., Hoffland E., Mulder C., de Goede R.G.M. (2015). Effects of GM potato Modena on soil microbial activity and litter decomposition fall within the range of effects found for two conventional cultivars. Biol. Fertil. Soils.

[105] Sohn S.-I., Oh Y.-J., Kim B.-Y., Kweon S.-J., Cho H.-S., Ryu T.-H. (2015). Effect of genetically modified rice producing resveratrol on the soil microbial communities. J. Korean Soc. Appl. Biol. Chem..

[106] Debruyn J.M., Bevard D.A., Essington M.E., Mcknight J.Y., Schaeffer S.M., Baxter H.L., Mazarei M., Mann D.G.J., Dixon R.A., Chen F. (2017). Field-grown transgenic switchgrass (Panicum virgatum L.) with altered lignin does not affect soil chemistry, microbiology, and carbon storage potential. GCB Bioenergy.

[107] Xu S., Ottinger S.L., Schaeffer S.M., DeBruyn J.M., Stewart C.N., Mazarei M., Jagadamma S. (2019). Effects of field-grown transgenic switchgrass carbon inputs on soil organic carbon cycling. PeerJ..

[108] Chen Y., Pan L., Ren M., Li J., Guan X., Tao J. (2022). Comparison of genetically modified insect-resistant maize and non-transgenic maize revealed changes in soil metabolomes but not in rhizosphere bacterial community. GM Crops Food.

[109] Lakshmanan V., Bais H., Sherrier J. (2015). Root microbiome assemblage is modulated by plant host factors. Plant Microbe Interactions.

[110] Ng E.L., Patti A.F., Rose M.T., Schefe C.R., Wilkinson K., Cavagnaro T.R. (2014). Functional stoichiometry of soil microbial communities after amendment with stabilized organic matter. Soil Biol. Biochem..

[111] Zi H., Hu L., Wang C. (2022). Differentiate responses of soil microbial community and enzyme activities to nitrogen and phosphorus addition rates in an alpine meadow. Front. Plant Sci..

[112] Choi Y.I., Noh E.W., Choi K.S. (2009). Low level expression of prokaryotic tzs gene enhances growth performance of transgenic poplars. Trees.

[113] Nam K.J., Kim D.Y., Nam K.-H., Pack I.S., Park J.H., Jeong S.-C., Choi Y.I., Noh E.W., Kim C.-G. (2014). Effects of transgenic poplars expressing increased levels of cellular cytokinin on rhizosphere microbial communities. Eur. J. Soil Biol..

[114] Saxena D., Stotzky G. (2001). Bt corn has a higher lignin content than non-Bt corn. Am. J. Bot..

[115] Wu W.-X., Ye Q.-F., Min H., Duan X.-J., Jin W.-M. (2004). Bt-transgenic rice straw affects the culturable microbiota and dehydrogenase and phosphatase activities in a flooded paddy soil. Soil Biol. Biochem..

[116] Huang S., Zeng X., Wei Y., Bai X., Jin Z., Zhang M., Wang Z., Wang H., Qu J., Di H. (2020). Decomposition of betaine aldehyde dehydrogenase transgenic maize straw and its effects on soil microbial biomass and microbiota diversity. Appl. Soil Ecol..

[117] Hannula S.E., de Boer W., Baldrian P., van Veen J.A. (2013). Effect of genetic modification of potato starch on decomposition of leaves and tubers and on fungal decomposer communities. Soil Biol. Biochem..

[118] Hopkins D.W., Webster E.A., Chudek J.A., Halpin C. (2001). Decomposition of stems from tobacco plants with genetic modifications to lignin biosynthesis. Soil Biol. Biochem..

[119] Tilston E.L., Halpin C., Hopkins D.W. (2004). Genetic modifications to lignin biosynthesis in field-grown poplar trees have inconsistent effects on the rate of woody trunk decomposition. Soil Biol. Biochem..

[120] Deacon L.J., Pryce-Miller E.J., Frankland J.C., Bainbridge B.W., Moore P.D., Robinson C.H. (2006). Diversity and function of decomposer fungi from a grassland soil. Soil Biol. Biochem..

[121] Vauramo S., Pasonen H.-L., Pappinen A., Setala H. (2006). Decomposition of leaf litter from chitinase transgenic silver birch (Betula pendula) and effects on decomposer populations in a field trial. Appl. Soil Ecol..

[122] Qin W., Hu C., Oenema O. (2015). Soil mulching significantly enhances yields and water and nitrogen use efficiencies of maize and wheat: A meta-analysis. Sci. Rep..

[123] Marral M.W.R., Khan M.B., Ahmad F., Farooq S., Hussain M. (2020). The influence of transgenic (Bt) and nontransgenic (non-Bt) cotton mulches on weed dynamics, soil properties and productivity of different winter crops. PLoS ONE.

[124] Dellero Y. (2020). Manipulating amino acid metabolism to improve crop nitrogen use efficiency for a sustainable agriculture. Front. Plant Sci..

[125] Lebedev V.G., Popova A.A., Shestibratov K.A. (2021). Genetic engineering and genome editing for improving nitrogen use efficiency in plants. Cells.

[126] Baker A., Ceasar S.A., Palmer A.J., Paterson J.B., Qi W., Muench S.P., Baldwin S.A. (2015). Replace, reuse, recycle: Improving the sustainable use of phosphorus by plants. J. Exp. Bot..

[127] Teng W., He X., Tong Y.-P. (2017). Transgenic approaches for improving use efficiency of nitrogen, phosphorus and potassium in crops. J. Integr. Agric..

[128] Iqrar S., Ashrafi K., Kiran U., Fatima S., Kamaluddin, Abdin M.Z., Kiran U., Abdin M.Z., Kamaluddin (2020). Chapter seven—Transgenic plants with improved nutrient use efficiency. Transgenic Technology Based Value Addition in Plant Biotechnology.

[129] Singer S.D., Soolanayakanahally R.Y., Foroud N.A., Kroeber R. (2020). Biotechnological strategies for improved photosynthesis in a future of elevated atmospheric CO_2_. Planta.

[130] Araus J.L., Sanchez-Bragado R., Vicente R. (2021). Improving crop yield and resilience through optimization of photosynthesis: Panacea or pipe dream?. J. Exp. Bot..

[131] Weir T.L., Park S.W., Vivanco J.M. (2004). Biochemical and physiological mechanisms mediated by allelochemicals. Curr. Opin. Plant Biol..

[132] Schmutterer H., Singh R.P., Schmutterer H. (2002). List of insect pests susceptible to neem products. The Neem Tree: Azadirachta Indica A. Juss and Other Meliacious Plants—Sources of Unique Natural Products for Integrated Pest Management, Medicine, Industry and Other Purposes.

[133] Gopal M., Gupta A.V., Arunachalam V., Magu S.P. (2007). Impact of azadirachtin, an insecticidal allelochemical from neem on soil microflora, enzyme and respiratory activities. Bioresour. Technol..

[134] Gu Y., Wang P., Kong C.H. (2009). Urease, invertase, dehydrogenase and polyphenoloxidase activities in paddy soil influenced by allelopathic rice variety. Eur. J. Soil Biol..

[135] Reigosa M.S., Gonzalesy L., Souto X.C., Pastoriza J.E., Narwal S.S., Hoagland R.E., Dilday R.H., Reigosa M.J. (2000). Allelopathy in forest ecosystem. Allelopathy in Ecological Agriculture and Forestry.

[136] Lebedev V.G., Krutovsky K.V., Shestibratov K.A. (2019). Fell Upas sits, the hydra-tree of death, or the Phytotoxicity of trees. Molecules.

[137] ISAAA (2022). ISAAA’s GM Approval Database. http://www.isaaa.org/gmapprovaldatabase/.

[138] Kikuchi A., Yu X., Shimazaki T., Kawaoka A., Ebinuma H., Watanabe K.N. (2009). Allelopathy assessments for the environmental biosafety of the salt-tolerant transgenic Eucalyptus camaldulensis, genotypes codA12-5B, codA12-5C, and codA20C. J. Wood Sci..

[139] Yu X., Kikuchi A., Shimazaki T., Yamada A., Ozeki Y., Matsunaga E., Ebinuma H., Watanabe K.N. (2013). Assessment of the salt tolerance and environmental biosafety of Eucalyptus camaldulensis harboring a mangrin transgene. J. Plant Res..

[140] Tran N.-H.T., Oguchi T., Matsunaga E., Kawaoka A., Watanabe K.N., Kikuchi A. (2021). Evaluation of potential impacts on biodiversity of the salt-tolerant transgenic Eucalyptus camaldulensis harboring an RNA chaperonic RNA-Binding-Protein gene derived from common ice plan. Transgenic Res..

[141] Shimazaki T., Kikuchi A., Matsunaga E., Nanto K., Shimada T., Watanabe K.N. (2009). Establishment of a homogenized method for environmental biosafety assessments of transgenic plants. Plant Biotechnol..

[142] Lebedev V., Larionova A., Bykhovets S., Shanin V., Komarov A., Shestibratov K. Model assessment of transgenic trees impact on nitrogen and carbon cycles in forest plantations. Proceedings of the IUFRO Tree Biotechnology Conference 2015 “Forests: The importance to the planet and society”.

